# Assessing competitiveness and complementarity in agricultural trade between China and Cambodia pre-pandemic and post-pandemic

**DOI:** 10.1371/journal.pone.0321081

**Published:** 2025-04-02

**Authors:** Zhe Tao, Siva Shankar Ramasamy, Nathee Naktnasukanjn, Fangli Ying

**Affiliations:** 1 International College of Digital Innovation, Chiang Mai University, Chiang Mai, Thailand; 2 Wuxi Taihu University, Wuxi, Jiangsu, China; Thammasat University, THAILAND

## Abstract

The concepts of trade competitiveness and complementarity function as key indicators for assessing the strengths and weaknesses of a nation’s export profile, as well as predicting future trade trajectories. Analyzing the levels of competitiveness and complementarity within the agricultural sectors of Cambodia and China is imperative for understanding and comparing their respective competitive advantages. This study utilizes quantitative research methodologies to analyze the agricultural trade dynamics between China and Cambodia before and after the pandemic. Specifically, the research involves the calculation of the revealed comparative advantage (RCA) index, the trade complementarity index (TCI), the trade intensity index (TII), and the Grubel-Lloyd (GL) index, covering the period from 2017 to 2022. The data is from the UN Commodity Trade Statistics Database through the HS code 1-24. The study used a three-year period as a short research phase and applied two phases to compare pre-pandemic and post-pandemic. The empirical results demonstrated that China and Cambodia both exhibit a strong comparative advantage in exporting specific items. From 2017 to 2019, China has comparative advantages in exporting HS items 05,13 and 16, and Cambodia has comparative advantages in exporting HS items 10,11 and 17. From 2020 to 2022, China has a comparative advantage in exporting HS item 13, and Cambodia has a comparative advantage in exporting HS item 10. Both China and Cambodia have specific complementary agricultural items in bilateral agricultural exports from 2017 to 2019, but China does not have complementary exports in its exports and Cambodia’s imports from 2020 to 2022. The trade intensity index underscores the advantages of their bilateral trade in the direction of China’s exports to Cambodia. Inter-industry advantages are identified in specific agricultural products. This study is beneficial for the Cambodian government and relevant stakeholders in formulating sustainable policies to promote agricultural trade after pandemic. The insights derived from these indices will furnish a rigorous foundation upon which the Cambodian government can develop and implement evidence-based sustainable strategies to optimize and enhance its agricultural trade performance.

## 1. Introduction

The competitiveness and complementarity of agricultural trade are considered crucial scientific factors for policy decision-making. In recent years, many countries have aimed to enhance bilateral trade to strengthen their economies. According to Cambodian government statistics, the country’s Gross Domestic Product (GDP) in 2023 was approximately $32.17 billion, marking a 5.6% increase from the previous year. The per capita GDP stood at $1,917. The industrial sector experienced a 5% growth, the service sector grew by 8.1%, and the agricultural sector saw a 0.9% increase. Additionally, a recent report from the General Department of Customs and Excise of Cambodia revealed that the nation’s international trade volume reached $46.8 billion in 2023, with China remaining its primary agricultural trading partner [1,2]. Analyzing the competitiveness and complementarity of agricultural trade between China and Cambodia holds significant potential for positively informing Cambodia’s future trade policy formulation. Additionally, studying the competitiveness and complementarity of agricultural trade between China and Cambodia offers further insights that are applicable to the analysis of agricultural trade between China and ASEAN countries within the framework of the Belt and Road Initiative. From 2010 to 2019, the trade volume of agricultural products between China and Cambodia grew significantly, increasing from $26.255 million to $360 million, with an average annual growth rate of 33.5%. During this period, China’s exports of agricultural products to Cambodia rose from $22.029 million to $90.854 million, reflecting an average annual growth rate of 17.1%. Simultaneously, China’s imports of agricultural products from Cambodia surged from $4.226 million to $260 million, achieving an impressive average annual growth rate of 58.3% [3]. Cambodia occupies a strategically crucial position in China’s efforts to enhance its influence within Southeast Asia. It serves as a vital component in China’s broader strategy to counterbalance and mitigate the influence of longstanding geopolitical rivals, while also potentially challenging and diminishing the dominance of American hegemony in the region [4].

The agricultural sector plays a crucial role in the trade relationship between China and Cambodia. Given their geographic proximity and political alignment, the two countries have established a stable and long-lasting trade partnership. Comparative advantages are crucial in shaping effective trade policies. The primary task is to ascertain the levels of competitiveness and complementarity in agricultural trade between Cambodia and China before and after the pandemic.

## 2. Literature review

In previous studies, the concepts of competitiveness and complementarity in agricultural trade between nations have emerged as critical areas of inquiry within global economic discourse. Sok et al. highlighted the dual nature of Cambodia’s engagement with the Belt and Road Initiative (BRI), noting both the benefits and challenges it poses to the country’s social development and economic growth. While the BRI has substantially advanced infrastructure projects, foreign direct investments (FDIs), and trade, it has also raised concerns regarding the efficacy of aid and the potential risks of debt dependency [5]. Neak and Sok indicated that the Cambodia-China Free Trade Agreement, with the objective of augmenting the annual bilateral trade volume between the two nations to US$ 10 billion, encompasses a comprehensive range of aspects including goods, investment, technical cooperation, e-commerce, and Belt and Road Initiative (BRI) cooperation [6]. China’s investment might also enforce the development of Cambodia’s agriculture. For instance, Significant Chinese investment has been made in Cambodia’s rice sector, involving elements such as rice mills and irrigation systems. These investments constitute a segment of more extensive trade agreements, especially those centered around jasmine rice. Through such agreements, Cambodian agriculture has been incorporated into the nascent Chinese food regime. This assimilation has led to an escalating dependence of Cambodian farmers on export commodity markets, which are characterized by instability due to the variability in market demands and prices [7]. By identifying the competitive and complementary relationships in agricultural product trade, the two countries can make investments in specific products and introduce relevant policies, thereby promoting trade between them. Countries involved in the BRI, alongside China, should deepen agricultural trade cooperation by utilizing existing bilateral and multilateral frameworks to support development [8]. For example, Zhang employed the RCA index to examine agricultural trade data between China and Brazil, thereby identifying the comparative advantages of agricultural products in each nation [9]. This body of work underscores the ongoing scholarly efforts to refine methodologies for assessing competitive and complementary trade dynamics between countries. The concepts of complementarity and competitiveness are foundational to international trade cooperation [10]. In their analysis of bilateral trade between China and ASEAN countries, Zhang and Xu identified significant similarities in the export trade structures of China and ASEAN, suggesting that their trade relationships are characterized more by competition than complementarity. Their findings also revealed that China is at a considerable disadvantage in the trade of primary products relative to ASEAN countries [11]. Sang and Yang explored the competitiveness and complementarity of China and its “Belt and Road” partners, offering insights into specific trade relationships and informing more effective policy-making for China. Their research demonstrated a strong trade complementarity between Southeast Asian countries and China [12].

Furthermore, Zhu and Chen suggested that understanding the competitiveness and complementarity of agricultural trade could be foundational for shaping trade policies and developing countries’ potential through comparative advantages, as seen in the case of India and China [13]. Similarly, Tao et al. found that research on these aspects could enhance trade effectiveness between Malaysia and China. They used four methods, including the RCA index, trade complementarity index, trade intensity index, and Grubel-Lloyd index, to analyze the agricultural trade between China and Malaysia. These studies collectively indicate that investigating competitiveness and complementarity is a preferred method for improving international trade performance [14].

Scholars frequently utilize traditional economic theories as foundational frameworks to analyze competitiveness. Within this context, competitiveness is linked to the concept of absolute advantage, as articulated by Smith [15], and Ricardo’s principle of comparative advantage [16]. These classical economic theories have spurred the development of various methodologies for assessing trade effects within specific sectors. A notable example is Balassa’s [17] revealed comparative advantage (RCA) index, which provides an empirical measure of a country’s export performance relative to a defined group of countries within the same industry. The RCA index facilitates the evaluation of comparative advantage by benchmarking a country’s export performance against that of its peers in the same sector. However, the utility of this method is constrained by its dependence on specific underlying assumptions.

Balassa further illustrated that the RCA index could effectively capture the trade performance of individual countries, particularly in the context of manufactured goods [17]. Building on this foundation, Laursen revisited Balassa’s RCA, proposing the “revealed symmetric comparative advantage” (RSCA) index. Laursen argued that the RSCA index offers a more nuanced representation of a country’s economic activities, although its applicability remains confined to more narrowly defined economic sectors [18]. Lv corroborated the efficacy of the RCA index as a robust tool for analyzing trade structures and informing policy, highlighting its value in macroeconomic analysis [19]. Consequently, the RCA index is widely regarded as an effective approach for assessing trade performance and guiding macroeconomic policy decisions.

Intra-industry and inter-industry advantages in agricultural research are critical factors for comparing mutual advantages between countries. Grubel and Lloyd introduced the Grubel-Lloyd (GL) index to analyze intra-industry trade and evaluate intra-trade and inter-trade advantages [20]. Feng highlighted that Chinese scholars primarily focus on the intra-industry trade situation, influencing factors, bilateral trade dynamics, the impact of zero tariffs on China’s economy, and the effects of trade barriers in their studies on China’s agricultural trade with ASEAN [21].

Azhar et al. argued that the GL index is the most appropriate measure for documenting an industry’s trade patterns over a specific period [22]. They also proposed a method to measure intra-industry trade based on the import-export ratio, which captures changes in both relative and absolute intra-industry trade (IIT), regardless of trade magnitude and direction. Previous studies have demonstrated the method’s effectiveness. For instance, Fan and Li conducted an empirical study of intra-industry trade of agricultural products between China and ASEAN from 2001 to 2010 using the GL index. Their findings indicated that the level of intra-industry trade between China and ASEAN was low, with trade increases mainly driven by inter-industry trade [23]. Therefore, the Grubel-Lloyd index has proven to be an effective tool for researchers to analyze the advantages of inter-industry and intra-industry.

The trade complementarity index (TCI) can effectively measure the degree of product matching between export supply and import demand in bilateral trade, with complementarity influenced by industrial structure, consumer demand, and endowment factors [24]. Hoang utilized the TCI to examine agricultural trade complementarity within ASEAN from 1997 to 2015, revealing that ASEAN’s agricultural export patterns weakly align with regional import demands [25].

Drysdale advanced the intensity analysis by decomposing it into “commodity bias” or “the degree of complementarity”[26]. Yamazawa proposed an integrated analytical framework combining trade intensity analysis, comparative advantage theory, and the trade gravity model within the trade intensity index (TII) model [27]. Zhang and Tang used the TII model to assess trade potential and closeness, further analyzing trade complementarity, and found that the Belt and Road Initiative significantly impacts China’s exports with its participating countries, underscoring the influence of relative basis factors on trade intensity [28]. Economic integration policies are also effective in promoting regional trade and national competitiveness [29].

Agricultural trade policies may be influenced by various competitive factors unique to each country. Scholars have provided a theoretical foundation for macroeconomic policy development, which serves as a reference for policymakers. Introducing competitive considerations into trade defense policies is recommended [30]. Furthermore, developing countries should prioritize policy design focused on human capital development, property rights protection, and fair competition to enhance economic competitiveness [31].

## 3. Method

The study examines agricultural data from 2017 to 2022 from the UN Commodity Trade Statistics Database [32], employing the theories of comparative advantage and complementary trade, the trade intensity approach, and the Grubel-Lloyd index method. The study used a three-year period as a short research phase and applied two phases to compare pre-pandemic and post-pandemic. However, short-term data can only reflect corresponding results within a short timeframe and cannot present long-term phased data outcomes. Due to the impact of the pandemic and the time limitations of the comparison period, the experiment uses short-term data as a basis, assuming that short-term data can provide effective results.

### 3.1 Method 1

The distinct comparative advantages in agricultural exports between China and Cambodia can be attributed to variations in industrialization, agricultural practices, natural environments, and political structures. The evaluation of these comparative advantages is grounded in the framework provided by the Japan External Trade Organization (JETRO) for analyzing the revealed comparative advantage (RCA) index [33]. This study utilizes the RCA index [17] as a methodological tool to systematically assess and compare the relative strengths and weaknesses of agricultural exports from both nations.


$$RCA_{i}^{k}=\frac{X_{i}^{k}/X_{\omega }^{k}}{X_{i}^{t}/X_{\omega }^{t}}$$ (1)

*X*_*i*_^*k*^ and *X*_*ω*_^*k*^ are the value of *k* commodities from *i* country exporting to the world and the value of all *k* commodities exported to the world. *X*_*i*_^*t*^ and *X*_*ω*_^*t*^ are the value of country *i* ‘s total exports to the world and all commodities exported to the world market.

*RCA*_*i*_^*k*^ is the revealed comparative advantage index indicating export *k* in country *i*. *RCA*_*i*_^*k*^ ≥  2.5 means an extreme competitive advantage; 1.25 ≤  *RCA*_*i*_^*k*^ <  2.5 means a strong competitive advantage; 0.8 ≤  *RCA*_*i*_^*k*^ <  1.25 means a medium competitive advantage; *RCA*_*i*_^*k*^ <  0.8 means a weak competitive advantage. The RCA index is used to study the comparative advantage of specific product categories in agricultural trade between China and Cambodia.

### 3.2 Method 2

This study examines the agricultural trade complementarity between China and Cambodia through the application of trade complementarity theory. Utilizing the trade complementarity index (TCI), the analysis focuses on the complementary dynamics of trade flows between the two countries, with particular emphasis on assessing the complementarity of products exported from one nation to the other. The TCI is calculated by:


$$RCA_{i}^{k}=\frac{X_{i}^{k}/X_{\omega }^{k}}{X_{i}^{t}/X_{\omega }^{t}},$$ (2)

$$TCI_{ij}^{k}=RCA_{i}^{k}\cdot rca_{j}^{k},$$ (3)

$$rca_{j}^{k}=\frac{y_{j}^{k}/y_{w}^{k}}{y_{j}^{t}/y_{w}^{t}},$$ (4)

*RCA*_*i*_^*k*^ is a comparative advantage index of country *i* ‘s *k* commodity exports, *X*_*i*_^*k*^ and *X*_*ω*_^*k*^ are the value of *k* commodities exported from country *i* to the world, and all *k* commodities’ value exported to the world. *X*_*i*_^*t*^ and *X*_*ω*_^*t*^ are the total export value from country *i* to the world and all commodities’ value exported to the world market. *rca*_*j*_^*k*^ is a competitive disadvantage index of *j* country’s *k* commodity imports. *y*_*j*_^*k*^ and *y*_*w*_^*k*^ are all *k* commodity’s value imported to *j* country and all *k* commodity’s value imported to the world. *y*_*j*_^*t*^ and *y*_*w*_^*t*^ are all import value to *j* country and all import value to the world. In the method, the world’s total import value of *A* products equals the world’s total export value of *A* products.

TCI > 1 means the two countries have strong complementarity, and TCI < 1 means the two countries have weak complementarity. The TCI index is used to study the complementary advantage of specific product categories in agricultural trade between China and Cambodia.

### 3.3 Method 3

The trade intensity index (TII) approach is utilized to analyze bilateral trade flows and assess the closeness of trade relationships between countries, with a higher TII indicating a closer trade relationship. Kojima enhanced the TII method [34], and Drysdale [26] further refined it by introducing two determinants: special country bias and commodity bias. The special country bias accounts for the effects of geography, politics, history, and institutions on international trade. Brown [35] and Kojima [34] determined that a higher trade intensity index (TII) value, particularly greater than 1, indicates more favorable factors and stronger bilateral trade relations between countries.

The formula is given by:


$$T_{ij}=\left(x_{ij}/X_{it}\right)/\left(x_{wj}/X_{wt}\right),$$ (5)

where *x*_*ij*_ means the country *i*’s export value to country *j*, *X*_*it*_ means the country *i*’s total export value, *x*_*wj*_ means the *j*’s total import value, and *X*_*wt*_ means the world’s total import value.

*T*_*ij*_ >  1 indicates that there are significant positive factors on bilateral trade flow, and the bigger number means a better positive effect. *T*_*ij*_ <  1 indicates an insignificant positive factor on bilateral trade flow, the lower number means the worse trade effect on trade relationship. In the method, the assumption is that the world’s total import value of *A* products equals the world’s total export value of *A* products. The TII index can effectively identify the closeness of agricultural trade between China and Cambodia.

### 3.4 Method 4

Grubel and Lloyd introduced the Grubel-Lloyd index as a methodological tool for the analysis of intra-industry trade. This index allows for a more nuanced understanding of trade patterns by quantifying the extent to which exports and imports occur within the same industry [20]. The Grubel-Lloyd index method was implemented to analyze intra-industry trade, and the formula is as follows:


$$GL_{j}=1-\frac{\left|X_{j}-M_{j}\right|}{X_{j}+M_{j}},$$ (6)

*X*_*j*_ means the country *i*’s export value to country *k*, *M*_*j*_ is the country *i*’s import value from country *k*, and *j* is the targeted category of the product industry.

If *GL*_*j*_ is close to 1, the agricultural product in this research is in intra-industry trade advantage; if *GL*_*j*_ is close to 0, the agricultural product in this research is in inter-industry trade advantage. The GL index can effectively identify which categories in agricultural trade between China and Cambodia have inter-industry trade advantages and which have intra-industry trade advantages.

## 4. Results

The data presented in Tables 2 to 9 reflect the outcomes of the methodologies applied based on the HS Code Classification (Table 1). These results offer a robust quantitative representation of the calculated data by effectively comparing and contrasting the findings. However, the validity of these results is contingent upon specific hypotheses that other factors will not affect the final results, and their actual significance can only be accurately interpreted within these constraints. Given the numerous uncertainties inherent in this study, the interpretation of the results is necessarily limited to certain analytical paths.

**Table 1:** HS Code Classification of Agricultural Products.

HS Code	Meaning
01	Animals; live
02	Meat and edible meat offal
03	Fish and crustaceans, molluscs and other aquatic invertebrates
04	Dairy produce; birds’ eggs; natural honey; edible products of animal origin, not elsewhere specified or included
05	Animal originated products; not elsewhere specified or included
06	Trees and other plants, live; bulbs, roots and the like; cut flowers and ornamental foliage
07	Vegetables and certain roots and tubers; edible
08	Fruit and nuts, edible; peel of citrus fruit or melons
09	Coffee, tea, mate and spices
10	Cereals
11	Products of the milling industry; malt, starches, inulin, wheat gluten
12	Oil seeds and oleaginous fruits; miscellaneous grains, seeds and fruit, industrial or medicinal plants; straw and fodder
13	Lac; gums, resins and other vegetable saps and extracts
14	Vegetable plaiting materials; vegetable products not elsewhere specified or included
15	Animal or vegetable fats and oils and their cleavage products; prepared animal fats; animal or vegetable waxes
16	Meat, fish or crustaceans, molluscs or other aquatic invertebrates; preparations thereof
17	Sugars and sugar confectionery
18	Cocoa and cocoa preparations
19	Preparations of cereals, flour, starch or milk; pastrycooks’ products
20	Preparations of vegetables, fruit, nuts or other parts of plants
21	Miscellaneous edible preparations
22	Beverages, spirits and vinegar
23	Food industries, residues and wastes thereof; prepared animal fodder
24	Tobacco and manufactured tobacco substitutes

Source: The United Nations Commodity Trade Statistics Database [32].

Table 2 provides evidence that, from 2017 to 2019, China exhibited a robust comparative advantage in the export of items 05, 13, and 16, with each of their respective revealed comparative advantage (RCA) indices exceeding 1.25. The overall RCA values for China during this period were 0.3714 in 2017, 0.3747 in 2018, and 0.3598 in 2019, resulting in a three-year average RCA index of 0.3686. Similarly, Cambodia demonstrated a strong comparative advantage in the export of items 10, 11, and 17, with RCA indices consistently surpassing 1.25. Notably, item 10 in Cambodia revealed an exceptionally strong comparative advantage, with RCA indices exceeding 2.5. Cambodia’s RCA values over the same period were 0.5910 in 2017, 0.6608 in 2018, and 0.5675 in 2019, culminating in a three-year average RCA index of 0.6064.

**Table 2:** Revealed comparative advantage (RCA) index of Cambodia’s and China’s agricultural exports (2017, 2018, and 2019).

HS code	RCA Index of China’s Agricultural Export			RCA Index of Cambodia’s Agricultural Export		
	2017	2018	2019	2017	2018	2019
01	0.1969	0.1803	0.1662	0.0544	0.0332	0.0115
02	0.0567	0.0517	0.0459	0.0007	0.0004	0.0000
03	0.8800	0.8372	0.7741	0.0080	0.0098	0.0039
04	0.0523	0.0502	0.0461	0.0102	0.1614	0.0643
05	1.7519	1.7091	1.5995	0.0147	0.0057	0.0047
06	0.1268	0.1320	0.1435	0.0015	0.0009	0.0029
07	1.1772	1.1256	1.0407	0.3892	0.3207	0.0461
08	0.3451	0.3291	0.3586	0.1294	0.3179	0.6181
09	0.4415	0.5321	0.5506	0.3425	0.2680	0.2812
10	0.0492	0.0646	0.0750	5.0373	5.4754	4.7554
11	0.2405	0.3036	0.3060	2.2489	2.5975	1.7398
12	0.2064	0.2074	0.2230	0.0226	0.0330	0.0149
13	1.4204	1.4277	1.4297	0.0461	0.0535	0.0409
14	1.0383	1.0414	0.8979	0.0000	0.0000	0.0121
15	0.0651	0.0897	0.0990	0.3931	0.3881	0.4006
16	1.4283	1.4822	1.3248	0.0000	0.0000	0.0000
17	0.2763	0.3334	0.3478	2.3727	2.9361	2.0836
18	0.0619	0.0640	0.0599	0.0269	0.0360	0.0232
19	0.1787	0.1979	0.2083	0.1008	0.1312	0.1935
20	0.9661	0.9543	0.9117	0.0127	0.1953	0.3698
21	0.3578	0.3628	0.3728	0.0047	0.0112	0.0092
22	0.1485	0.1451	0.1241	0.2667	0.1315	0.1478
23	0.2917	0.3017	0.2731	0.4140	0.4236	0.2208
24	0.2532	0.2416	0.2361	0.9947	1.0963	0.8496
Total	0.3714	0.3747	0.3598	0.5910	0.6608	0.5675

Source: Calculated results from data of the United Nations Commodity Trade Statistics Database.

These findings indicate that both China and Cambodia possess significant comparative advantages in the export of specific products. However, when examining the overall RCA indices, China’s three-year average of 0.3686 is lower than Cambodia’s 0.6064, and Cambodia demonstrated a 64.5% higher average RCA index than China during this period, indicating a stronger comparative advantage in agricultural trade on a broader scale. Additionally, the results reveal that China and Cambodia exhibit complementary advantages in the export of particular items, highlighting potential areas for mutually beneficial trade cooperation.

Turning to Table 3, the data from 2020 to 2022 reinforces these observations. During this period, China continued to demonstrate a strong comparative advantage in the export of item 13, with RCA indices of 0.3068 in 2020, 0.2861 in 2021, and 0.3056 in 2022, leading to a three-year average RCA index of 0.2995. In contrast, Cambodia maintained its strong comparative advantage in the export of item 10, with RCA indices exceeding 1.25 throughout the period. Particularly, item 10 in Cambodia displayed an exceptionally strong comparative advantage, with RCA values surpassing 2.5. Cambodia’s RCA indices for item 10 were 0.4890 in 2020, 0.6259 in 2021, and 0.5647 in 2022, resulting in a three-year average RCA index of 0.5599.

**Table 3:** Revealed comparative advantage (RCA) index of Cambodia’s and China’s agricultural exports (2020,2021 and 2022).

HS code	RCA Index of China’s Agricultural Products Export			RCA Index of Cambodia’s Agricultural Products Export		
	2020	2021	2022	2020	2021	2022
01	0.1806	0.1589	0.1547	0.0158	0.0435	0.0118
02	0.0351	0.0373	0.0392	0.0000	0.0000	0.0000
03	0.6615	0.5616	0.5883	0.0032	0.0043	0.0028
04	0.0425	0.0398	0.0412	0.0000	0.0107	0.0477
05	1.2139	1.1206	1.1247	0.0002	0.0292	0.0380
06	0.1402	0.1330	0.1446	0.0001	0.0000	0.0003
07	0.8441	0.7961	0.7936	0.0508	0.3878	0.0961
08	0.3552	0.2881	0.2661	0.9624	1.6656	1.8760
09	0.5198	0.4691	0.3871	0.2161	0.6226	0.4560
10	0.0534	0.0465	0.0403	3.8293	3.5762	2.7394
11	0.2287	0.1817	0.2512	1.1522	1.1216	1.3357
12	0.1761	0.1503	0.1364	0.0095	0.0048	0.0005
13	1.3817	1.4946	1.6411	0.0535	0.0414	0.0457
14	0.8312	0.9118	1.0532	0.0001	0.0000	0.0000
15	0.0944	0.1054	0.1392	0.3083	0.5050	0.4790
16	1.1545	1.3144	1.2424	0.0000	0.0000	0.0014
17	0.2649	0.2627	0.2934	1.2020	1.3468	1.1198
18	0.0470	0.0520	0.0532	0.0117	0.0066	0.0088
19	0.1657	0.1759	0.1663	0.1580	0.1921	0.2723
20	0.8007	0.7693	0.8423	0.4994	1.0768	1.2413
21	0.3623	0.3941	0.4155	0.0102	0.0271	0.0305
22	0.1132	0.0969	0.1032	0.0308	0.0101	0.0259
23	0.2417	0.2500	0.2312	0.2449	0.5232	0.5822
24	0.1263	0.1084	0.9577	0.3071	0.3080	0.1628
Total	0.3068	0.2861	0.3056	0.4890	0.6259	0.5647

Source: Calculated results from data of the United Nations Commodity Trade Statistics Database.

When comparing the overall RCA indices from 2020 to 2022, China’s average RCA index of 0.2995 is again lower than Cambodia’s 0.5599, and Cambodia’s advantage persisted, with its RCA average 86.9% higher than China’s, underscoring its sustained strength in agricultural trade relative to China. China’s 3-year average RCA decreased by 18.74% after pandemic, and Cambodia’s 3-year average RCA decreased by 7.7% after pandemic; the disparity in RCA decline suggests that Cambodia’s agricultural trade was less adversely affected by the pandemic compared to China’s. This might be due to the effect of China and Cambodia’s free trade agreement and China’s Belt and Road Initiative.

Table 4 indicates that, from 2017 to 2019, China’s exports and Cambodia’s imports of agricultural products are strongly complementary, mainly to item 24. China’s imports and Cambodia’s exports of agricultural products are strongly complementary mainly to items 10 and 11. The research shows that China and Cambodia are highly complementary in terms of these specific agricultural products. From the basis of Cambodia’s exports and China’s imports, the TCI of item 10 is extremely high, which means that the complementarity effect is extremely high on this item.

**Table 4:** TCI based on China’s exports and Cambodia’s imports, and Cambodia’s exports and China’s imports (2017, 2018, and 2019).

HS Code	TCI based on China’s exports to Cambodia			TCI based on Cambodia’s exports to China		
	2017	2018	2019	2017	2018	2019
01	0.1146	0.0553	0.0392	0.0085	0.0053	0.0022
02	0.0034	0.0038	0.0040	0.0005	0.0003	0.0000
03	0.0329	0.0187	0.0261	0.0052	0.0084	0.0045
04	0.0209	0.0235	0.0192	0.0057	0.0896	0.0404
05	0.0906	0.1960	0.2548	0.0084	0.0033	0.0032
06	0.0025	0.0092	0.0201	0.0002	0.0001	0.0003
07	0.1826	0.0984	0.0494	0.1016	0.0815	0.0087
08	0.0275	0.0274	0.0261	0.0657	0.2003	0.4990
09	0.0500	0.0641	0.0582	0.0299	0.0321	0.0488
10	0.0094	0.0275	0.0341	2.9069	2.5139	1.9322
11	0.9394	1.1677	1.3144	1.1213	1.3682	1.0085
12	0.0049	0.0049	0.0067	0.0963	0.1274	0.0555
13	0.5582	0.3450	0.7108	0.0152	0.0183	0.0186
14	0.0728	0.1196	0.0338	0.0000	0.0000	0.0144
15	0.0139	0.0168	0.0165	0.3102	0.3195	0.3984
16	0.3927	0.4089	0.3839	0.0000	0.0000	0.0000
17	0.3277	0.3305	0.3662	0.6439	0.8865	0.7502
18	0.0061	0.0032	0.0065	0.0036	0.0052	0.0034
19	0.2892	0.3777	0.4099	0.0762	0.1012	0.1576
20	0.1913	0.1344	0.1508	0.0021	0.0378	0.0773
21	0.4748	0.4238	0.4510	0.0015	0.0042	0.0038
22	0.4083	0.4013	0.3164	0.1155	0.0604	0.0616
23	0.9587	0.8950	0.7056	0.1894	0.1818	0.1025
24	1.9364	1.6253	1.2322	0.4107	0.3853	0.3234

Source: Calculated results from data of the United Nations Commodity Trade Statistics Database.

Table 5 indicates that, from 2020 to 2022, China’s exports and Cambodia’s imports of agricultural products are complementary to no items. China’s imports and Cambodia’s exports of agricultural products are strongly complementary mainly to item 10. The findings indicate that Cambodia and China possess complementary advantages in particular agricultural sectors. While 2017–2019 results exhibited a broader scope of complementarity (items 24, 10, and 11), 2020–2022 results narrowed the focus to item 10 alone. This shift could reflect structural changes in agricultural production, trade policies, or shifts in consumer demand in either country. Consequently, the implementation of targeted cooperative policies, such as building specific agricultural cooperation areas between nations, informed by these results, would likely enhance and strengthen bilateral trade relations between the two nations.

**Table 5:** TCI based on China’s exports and Cambodia’s imports, and Cambodia’s exports and China’s imports (2020,2021, and 2022).

HS Code	TCI based on China’s exports and Cambodia’s imports			TCI based on Cambodia’s exports and China’s imports		
	2020	2021	2022	2020	2021	2022
01	0.3731	0.1498	0.0441	0.0038	0.0144	0.0040
02	0.0040	0.0067	0.0114	0.0000	0.0001	0.0000
03	0.0348	0.0740	0.0643	0.0030	0.0038	0.0033
04	0.0221	0.0197	0.0198	0.0000	0.0082	0.0349
05	0.1567	0.4256	0.9154	0.0001	0.0217	0.0377
06	0.0168	0.0009	0.0022	0.0000	0.0000	0.0000
07	0.0405	0.0578	0.0560	0.0108	0.1087	0.0386
08	0.0253	0.0199	0.0302	0.7324	1.4927	2.0051
09	0.0546	0.0398	0.0408	0.0431	0.1450	0.0959
10	0.0308	0.0241	0.0105	2.4822	3.8195	2.6545
11	0.8307	0.5853	1.0296	0.6854	0.7559	1.0519
12	0.0065	0.0081	0.0069	0.0327	0.0182	0.0019
13	0.2660	0.3130	0.3652	0.0210	0.0170	0.0189
14	0.0637	0.0882	0.0510	0.0001	0.0000	0.0000
15	0.0159	0.0123	0.0157	0.2848	0.4451	0.3757
16	0.3175	0.3292	0.2983	0.0000	0.0000	0.0001
17	0.2848	0.3012	0.3463	0.6151	0.7140	0.6168
18	0.0043	0.0035	0.0048	0.0017	0.0010	0.0014
19	0.2962	0.2833	0.2596	0.1195	0.1217	0.1669
20	0.1280	0.1492	0.1609	0.0884	0.2104	0.2540
21	0.4325	0.4233	0.4292	0.0045	0.0112	0.0144
22	0.2817	0.1626	0.1495	0.0110	0.0039	0.0082
23	0.6753	0.7497	0.6804	0.1214	0.2868	0.3394
24	0.9081	0.4177	3.9737	0.0727	0.0848	0.0462

Source: Calculated results from data of the United Nations Commodity Trade Statistics Database.

Table 6 indicates that, from 2017 to 2019, the result showed that TII numbers are above 1 in the direction from China’s export to Cambodia, which means they have advantages in bilateral trade related to positive factors and the strong closeness of trade ties in this direction. The TII from Cambodia’s exports to China’s direction are <  1, and the advantages of trade closeness are weak. These factors might contain geographical factors, political factors, and local industrial construction factors [28]. Therefore, the two nations could enhance their agricultural trade collaboration by strengthening trade agreements and fostering industry partnerships, with a focus on specific export strategies tailored to mutual economic interests such as increasing the construction of transportation roads in both countries.

**Table 6:** Trade intensity index (TII) based on China’s export to Cambodia and Cambodia’s export to China (2017, 2018, and 2019).

Export direction base	China to Cambodia			Cambodia to China		
Year	2017	2018	2019	2017	2018	2019
Trade intensity index	2.55	2.62	2.90	0.84	0.96	0.86

Source: Calculated results from data of the United Nations Commodity Trade Statistics Database.

Table 7 indicates that from 2020 to 2022, the result showed that all of the TII numbers are above 1 in China’s export to Cambodia direction, which means they have advantages in bilateral trade related to positive factors and the strong closeness of trade ties in this direction. The TII from Cambodia’s exports to China’s direction are not above 1, and the advantages of trade closeness are weak. It means no matter before pandemic or after pandemic the closeness was not changed a lot. Therefore, the trade closeness between the two countries has not undergone significant changes due to the pandemic. China’s Belt and Road Initiative may play a role in promoting future trade development between China and Cambodia.

**Table 7:** Trade intensity index (TII) based on China’s export to Cambodia and Cambodia’s export to China (2020, 2021, and 2022).

Export direction base	China to Cambodia			Cambodia to China		
Year	2020	2021	2022	2020	2021	2022
Trade intensity index	2.80	2.94	3.03	0.70	1.00	0.76

Source: Calculated results from data of the United Nations Commodity Trade Statistics Database.

Table 8 demonstrates that, between 2017 and 2019, China and Cambodia exhibited comparative advantages in items 02, 04, 05, 06, 09, 10, 13, 14, 15, 16, 18, 21, and 22 at the inter-industry level, with the corresponding Grubel-Lloyd (GL) indexes for these items approaching 0. This indicates that China and Cambodia hold inter-industry advantages in these sectors in the short term. Conversely, neither country shows strong intra-industry advantages, as none of the GL indexes for the items approaches 1, suggesting an absence of significant intra-industry competitiveness in the short term. The results of this analysis offer a basis for constructing a trade framework that could reinforce either inter- or intra-industry advantages in specific sectors.

**Table 8:** Grubel-Lloyd index based on agricultural trade between China and Cambodia (2017, 2018, and 2019).

HS Code	GL Index		
	2017	2018	2019
01	0.0000	0.2615	0.0023
02	0.0000	0.0000	0.0000
03	0.0166	0.5763	0.0000
04	0.0000	0.0000	0.0000
05	0.0757	0.0000	0.0000
06	0.0000	0.0000	0.0000
07	0.8917	0.5065	0.3339
08	0.6732	0.3810	0.0306
09	0.0000	0.0035	0.0021
10	0.0000	0.0000	0.0005
11	0.4520	0.3944	0.9066
12	0.2569	0.7521	0.0089
13	0.0000	0.0000	0.0000
14	0.0000	0.0000	0.0000
15	0.0000	0.0000	0.0087
16	0.0000	0.0000	0.0000
17	0.1296	0.0517	0.2621
18	0.0378	0.0000	0.0198
19	0.3075	0.1014	0.1083
20	0.3307	0.4848	0.9829
21	0.0000	0.0009	0.0060
22	0.0002	0.0229	0.0751
23	0.4531	0.5239	0.9712
24	0.5566	0.4559	0.7148

Source: Calculated results from data of the United Nations Commodity Trade Statistics Database.

Similarly, Table 9 shows that from 2020 to 2022, China and Cambodia maintained inter-industry advantages in items 02, 03, 04, 05, 06, 08, 09, 10, 12, 13, 14, 15, 16, 21, and 22, as reflected by GL indexes that remain close to 0. This reinforces the conclusion that inter-industry competitiveness persists in the short term for these items. However, as in the earlier period, no items have GL indexes near 1, indicating a continued lack of strong intra-industry advantages. The overlapping set of items with inter-industry advantages across both periods underscores stable trade patterns between China and Cambodia in specific sectors. The insights from this method can be instrumental for stakeholders engaged in Cambodia-related trade, providing them with the strategic foundation to build a trade framework that capitalizes on inter- or intra-industry advantages in targeted product categories.

**Table 9:** Grubel-Lloyd index based on agricultural trade between China and Cambodia (2020, 2021, and 2022).

HS Code	GL Index		
	2020	2021	2022
01	0.0000	---	0.0000
02	0.0000	0.0000	0.0000
03	0.0000	0.0000	0.0000
04	0.0000	0.0000	0.0000
05	0.0000	0.0000	0.0006
06	0.0000	0.0000	0.0000
07	0.2247	0.3865	0.8923
08	0.0130	0.0558	0.0814
09	0.0006	0.0035	0.0001
10	0.0000	0.0000	0.0000
11	0.3425	0.4189	0.6915
12	0.0000	0.1979	0.0000
13	0.0000	0.0000	0.0000
14	0.0000	0.0000	0.0000
15	0.0000	0.0000	0.0000
16	0.0000	0.0000	0.0000
17	0.0247	0.0419	0.5444
18	0.0613	0.1004	0.4329
19	0.0329	0.0217	0.2899
20	0.7966	0.7003	0.2978
21	0.0357	0.0018	0.0000
22	0.1353	0.0271	0.0141
23	0.5992	0.6897	0.7646
24	0.9579	0.4461	0.8547

Source: Calculated results from data of the United Nations Commodity Trade Statistics Database.

## 5. Discussion

During the period from 2017 to 2019, China exhibited a robust comparative advantage in exporting items 05, 13, and 16, with RCA indices exceeding 1.25, indicating a strong comparative advantage in these agricultural products. The aggregate RCA indices for China in these years were 0.3714, 0.3747, and 0.3598, respectively, resulting in an average of 0.3686. However, a noticeable shift occurred from 2020 to 2022, where China’s comparative advantage became more concentrated, with item 13 remaining prominent while the overall RCA indices declined to 0.3068, 0.2861, and 0.3056, with a three-year average of 0.2995. This decline suggests a weakening of China’s overall comparative advantage in agricultural exports post-2019, potentially due to changing global trade dynamics, domestic policy adjustments, or external shocks such as the COVID-19 pandemic. Cambodia, however, experienced a slight decline in its comparative advantage. From 2017 to 2019, Cambodia showed a strong comparative advantage in items 10, 11, and 17, with RCA indices above 1.25 and item 10 with an RCA index over 2.5. The total RCA indices for these years were 0.5910, 0.6608, and 0.5675, averaging 0.6064. From 2020 to 2022, Cambodia maintained its comparative advantage in item 10, but the total RCA indices decreased slightly to 0.4890, 0.6259, and 0.5647, averaging 0.5599. This decline, albeit modest, suggests challenges in maintaining its export competitiveness in these items. China and Cambodia possess comparative advantages in the export of agricultural commodities. Cambodia exhibits a higher comparative advantage than China in the overall export of agricultural products from 2017 to 2022. The Cambodian government could adopt a strategic approach to agricultural product diversification, leveraging Cambodia’s comparative advantage in select agricultural commodities. By introducing targeted subsidies and providing technical support policies, the government can incentivize farmers and agribusinesses to enhance the production of these key products. Additionally, this strategy would foster the development of other agricultural products with strong export potential, thereby broadening Cambodia’s agricultural export base and reducing dependency on a limited range of commodities.

Cambodia’s trade relationship with China, however, exhibited a narrowing focus post-pandemic. From 2017 to 2019, strong complementarity existed in item 24 for China’s exports and Cambodia’s imports, while Cambodia’s exports and China’s imports were complementary mainly in items 10 and 11. This indicates targeted but strong trade relationships in these items. However, from 2020 to 2022, the complementarity narrowed to item 10 for Cambodia’s exports and China’s imports, with no strong complementary items identified for China’s exports and Cambodia’s imports. This suggests that the agricultural trade relationship between the two countries became more focused and potentially more dependent on fewer items post-pandemic.

The trade intensity index (TII) indicates that the trade relationship between China and Cambodia exhibits several favorable characteristics, with the trade value surpassing expectations given the two countries’ respective roles in global trade. This is particularly evident in the direction of China’s exports to Cambodia, where trade performance exceeds anticipated levels based on their global trade significance. The analysis of trade intensity indices (TII) over two periods, 2017-2019 and 2020-2022, reveals consistent patterns in the bilateral trade relationship between China and Cambodia. During both periods, the TII values for China’s exports to Cambodia remained above 1, indicating that China holds a significant advantage in this trade direction. This suggests strong trade ties and positive factors that facilitate China’s export dominance to Cambodia.

Conversely, the TII values for Cambodia’s exports to China were consistently not above 1 during both periods, highlighting weaker trade closeness and a limited advantage for Cambodia in exporting to China. The factors influencing these trends could include geographical proximity, political relationships, and local industrial development [28]. Belt and Road policy might also be a potential contributing factor [28]. It is essential to consider multiple relative foundational factors, such as the sustained positive political relations between the countries and their geographical advantages, which may also explain these outcomes. Based on trade intensity index (TII) data, it is imperative to enhance bilateral agricultural trade cooperation to capitalize on these advantages.

The results of the Grubel-Lloyd index analysis indicate that the two nations possess distinct agricultural commodities characterized by inter-industry advantages. Cambodia’s trade relationship with China is characterized by inter-industry advantages in items 02, 04, 05, 06, 09, 10, 13, 14, 15, 16, 18, 21, and 22 from 2017 to 2019, with no strong intra-industry advantages. This pattern continued into the 2020-2022 period, with a similar focus on inter-industry trade (items 02,03,04,05,06,08,09,10,12,13,14,15,16,21,22) and no significant intra-industry advantage. This consistency suggests that Cambodia’s agricultural trade strategy with China has remained stable over time, with a focus on leveraging its broader agricultural production capabilities rather than developing deep specialization in specific sectors. Natural agricultural advantages, local consumption demand, and agricultural industrialization could account for these differences. Adopting a more open and liberal trade policy may foster the growth of trade and enhance trade diversity. Nonetheless, implementing foreign exchange adjustment policies targeting specific sectors might benefit agricultural exports but could also lead to trade conflicts. According to a study by Chen et al., various factors, including product differentiation, economic scale, market structure, and foreign direct investment, significantly influence intra-industry development in China [36]. Economic scale, market structure, and foreign direct investment are positively correlated with intra-industry development. The Cambodian government needs to consider how to improve its intra-industry advantages over China. Given the distinct industrial structures and local policies in Cambodia and China, it is crucial for the Cambodian government to consider these differences when drawing on China’s policies for cooperation.

For further cooperation based on the results, researchers have proposed suggestions for leveraging comparative advantages. Wu’s research on trade complementarity and competitiveness among BRICS countries indicates that highly complementary products in different markets exhibit varying levels of competitiveness. These findings suggest potential policy directions. Policymakers must consider changes in the competitiveness of complementary products, even those with long-term complementary features [37]. Kea et al. revealed that Cambodia’s rice exports have become increasingly competitive over time. This enhancement in relative export competitiveness (REC) is attributed to the effective implementation of rice policies and the rectangular strategy [38]. The global value chain is expected to drive the global industry’s upgrade to a higher level [39]. This implies that governments should recognize the role of trade in foreign direct investment (FDI), services, and intermediate goods in promoting domestic industrial upgrading from a global perspective. Other policies, such as financial policies, could also be considered. Reduced financing costs resulting from low interest rates have the potential to enhance business activities and stimulate economic growth, thereby positively influencing the competitiveness of agricultural enterprises [40]. Cambodia should also contemplate cooperation with China and assess the side effects posed by other competitors and partners. The free trade agreement between China and Cambodia officially came into effect on January 1, 2022. Pursuant to the provisions of the agreement, most of the product categories within the goods trade between the two nations will be eligible for zero-tariff treatment. The impact of this policy may have had a positive effect on Cambodia’s agricultural trade in 2022 [41].

## 6. Conclusion

This research presents a comprehensive analysis of the complementarity and competitiveness in agricultural trade between Cambodia and China, focusing on the periods before and after the COVID-19 pandemic. The findings underscore that both countries possess distinct comparative advantages in exporting specific agricultural products, with Cambodia exhibiting a more pronounced comparative advantage in overall agricultural trade relative to China. Moreover, the study highlights particular categories of agricultural products that display complementary characteristics in export-import dynamics between the two nations. These results suggest that enhancing bilateral collaboration in agricultural trade could yield substantial mutual benefits, particularly through a targeted focus on areas where each country currently faces disadvantages. By implementing strategic policies, these trade weaknesses could potentially be converted into competitive strengths. The study further reveals that a higher trade intensity index indicates significant advantages arising from robust comparative advantage frameworks, favorable free trade agreements, and strategically advantageous geographic positions. However, the study also identifies variations in the benefits associated with inter-industry trade across different agricultural products. To maximize these trade benefits, the research suggests that adjustments in both domestic industrial policies and international trade strategies may be necessary. These findings offer valuable insights for policymakers aiming to foster a more synergistic and mutually beneficial agricultural trade relationship between Cambodia and China. Furthermore, this study has certain limitations, including the fact that factors such as climate change, technological advances, and multilateral trade dynamics were not considered as variables in this research.

There are some recommendations for further improvement in their agricultural trade. The enhancement of highway and railway networks between the two nations represents a pivotal strategy for improving the efficiency and convenience of transporting agricultural products from their points of origin to key trade hubs such as ports and airports. The construction of high-quality road infrastructure that links primary transportation arteries in key agricultural production zones could further minimize transportation time and reduce product losses, thereby strengthening the agricultural supply chain. Additionally, advancing the supporting infrastructure for cross-border e-commerce, particularly in the context of agricultural product trade, offers a means to enhance transaction efficiency and timeliness. Key measures in this domain include the development of cross-border payment systems, optimization of international logistics and distribution channels, and the establishment of overseas warehouses to facilitate seamless trade operations.To mitigate risks in agricultural trade, it is essential to develop a robust early-warning mechanism. Such a system would monitor international agricultural market dynamics, including price fluctuations, changes in trade barriers, and other relevant factors. Timely dissemination of warning information would enable stakeholders to devise preemptive strategies, thereby mitigating potential risks and enhancing resilience in agricultural trade. The Belt and Road Initiative (BRI) has played a transformative role in reducing trade costs between China and Cambodia, thereby fostering agricultural trade among countries along the BRI corridor. This initiative provides a platform for both nations to prioritize the production and export of agricultural goods in which they hold a comparative advantage, while simultaneously importing non-competitive products. Such an approach not only facilitates regional trade but also optimizes the utilization of economic and natural resources, removes trade barriers, reduces production subsidies, and enhances social welfare outcomes. Moreover, the establishment of a centralized database for traceability technology, particularly targeting agricultural exports to the Chinese market, is critical for compliance with rigorous standards related to origin, production processes, and processing methods. This centralized system enables the strategic alignment of agricultural production volumes with the competitive and complementary characteristics of specific products, ensuring a more adaptive and efficient production model. The stringent traceability requirements imposed by the Chinese market further provide Cambodian enterprises with a significant opportunity to cultivate and enhance the branding of their national agricultural products. This not only improves the competitiveness and reputation of Cambodian agricultural goods in international markets but also lays a foundation for sustainable growth in the sector by aligning production practices with global market demands.

Despite the valuable insights provided by this study, several limitations remain. Firstly, the findings are based on hypothetical scenarios, underscoring the need for further in-depth research to ascertain the practical applicability of these results to policy implementation. Secondly, the study’s reliance on short-term data limits its comprehensiveness. However, the inclusion of long-term data might introduce bias, as it may lack the comparative characteristics of pre- and post-pandemic conditions. Furthermore, the dynamic nature of international political and economic relations, as well as changes in the natural environment, could lead to unpredictable impacts on agricultural trade.
